# The Influence of Water Molecules on the π* Shape Resonances of the Thymine Anion

**DOI:** 10.1021/acs.jpca.5c01948

**Published:** 2025-05-12

**Authors:** Connor J. Clarke, E. Michi Burrow, Jan R. R. Verlet

**Affiliations:** 1 Department of Chemistry, 3057 Durham University, Durham DH1 3LE, United Kingdom; 2 J. Heyrovský Institute of Physical Chemistry, Czech Academy of Sciences, Dolejškova 3, Prague 8 18223, Czech Republic

## Abstract

Low-energy electrons have been shown to resonantly attach to DNA, inducing strand breakages and other damaging lesions. While computational studies have suggested that the nucleobase moieties can serve as the initial attachment site, there remains ambiguity over the exact character of the temporary anion resonances that form due to the unestablished role of the surrounding environment. Here, we investigate the influence of an aqueous environment on the low-lying anion shape resonances of the π* character of the thymine anion by applying frequency-resolved photoelectron spectroscopy to thymine-water cluster anions, T^–^(H_2_O)*_n_*, with an increasing degree of hydration, *n*. Our results indicate that spontaneous solvent rearrangement will stabilize the π_2_* and π_3_* states into bound electronic states, and we observe evidence for internal conversion to the anion ground state, further aiding long-term electron capture via these resonances.

## Graphical abstract

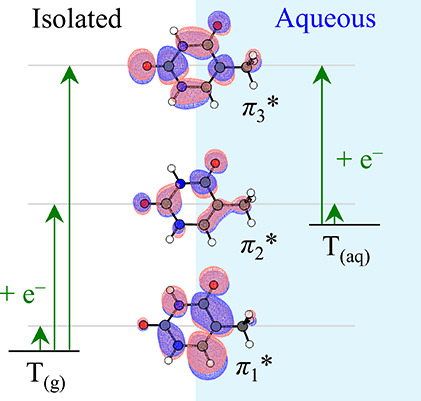

## Introduction

Ionizing radiation interacts with the components of biological cells to produce low-energy electrons in vast quantities (>10^4^ MeV^–1^).^1^ Such electrons have been extensively shown to induce damage to nearby DNA strands via the formation of transient negative ions (TNIs).^2–7^ It has been proposed that shape resonances with π* character on the nucleobases are the initial attachment sites for very low-energy (<4 eV) electrons, which subsequently couple to a σ* orbital along the phosphodiester C–O bond, instilling strand breakage.^8–16^ To identify which π* resonances are responsible, attempts have been made to correlate the lesion-inducing electron attachment energies to the resonant electron scattering energies of gas-phase nucleobases.^3,17^ However, such a straightforward comparison neglects the presence of the complex cellular environment surrounding DNA, which will inevitably influence the stability of the TNIs being formed.^18–22^ In particular, hydration stabilizes anionic molecules with respect to their neutral analogs,^23^ even if the anion exists in an excited state.^24–27^ Investigating nucleobases within an aqueous environment thereby offers a sensible route toward modeling more realistic electron-nucleobase interactions. For instance, low-energy (prehydrated) electrons have been shown to generate TNIs of aqueous pyrimidine nucleobases more efficiently than aqueous purine nucleobases.^28,29^ However, performing spectroscopy in the bulk aqueous phase sacrifices information on the electronic structure of TNIs that could elsewise be attainable with gas-phase experiments, and thus it remains unclear which π* states on the pyrimidine bases actively participate in the electron capture within an aqueous environment.

Anion-solvent clusters represent an appealing intermediary target. Cold clusters readily form *in vacuo* via supersonic expansion and can thus be interrogated using a range of gas-phase spectroscopic techniques.^30–32^ Solvation effects can then also be probed by incrementally increasing the size of the anion-solvent clusters being studied.^33,34^ As well-understood examples – for a large part through the work of Mark Johnson’s group – the effect of hydration on the binding of an excess electron or halide anions in water clusters has been characterized using IR action spectroscopy, which determines the hydrogen-bonding network in the clusters;^35–44^ UV–vis action spectroscopy to probe excited states;^45–47^ and photoelectron spectroscopy to track the incremental energetic stabilization.^23,48–54^ By measuring a sufficiently extensive range of clusters, physical properties emerging in the cluster regime can be projected into the bulk limit.^55^ However, starting from the anion does not access directly the TNIs that are involved in the initial electron capture due to a disparity in the equilibrium geometry of the cluster. Nonetheless, anion spectroscopy can provide valuable information on the π* states in the anion and some correlation to the same π* states of the TNIs can be diabatically drawn.

Building on such a cluster approach, we recently performed photoelectron spectroscopy on water clusters of the uracil (U) anion, i.e. U^–^(H_2_O)*_n_*, ranging from 1 ≤ *n* ≤ 35.^27,56^ As known previously,^57,58^ the electronic ground state of U^–^(H_2_O)*_n_* is the lowest energy π* state (labeled π_1_*, and originally identified in resonant electron scattering experiments^59^), which has been stabilized by the clustered water molecules. The electron binding energies of the anion clusters were determined and extrapolated to obtain the corresponding (adiabatic and vertical) detachment energies of U^–^_(aq)_. Excitation energies of the π_1_*→π_2_* and π_1_*→π_3_* transitions of U^–^(H_2_O)*_n_* (up to *n* = 10) were also obtained and showed that each of the three π* resonances of U are similarly stabilized via hydration. These measurements offer the energy differences in the geometry of the anions in an aqueous environment, and not those of the TNI initially formed, which is in the geometry of the neutral with its corresponding neutral environment. Some information concerning the connection between the two can be obtained in a simple Marcus picture, where the reorganization coordinate connecting the two is assumed to be harmonic. Such an approach offers the neutral-to-anion reorganization energy of aqueous U, and therefore also sheds light on the electron attachment energies to U_(aq)_ that populate each of the π* resonances. Measuring the positions of these resonances in such complex environments provides a critical step toward identifying which electronic states are initially populated in low-energy electron attachment to RNA. Here, we extend this methodology to the pyrimidine DNA nucleobase, thymine (T). Being a methylated form of U, T is expected to exhibit generally similar electron attachment properties. This study broadly confirms this expectation by examining thymine-water cluster anions, T^–^(H_2_O)*_n_*, but also highlights some subtle differences in the resonance positions and reorganization energies. Moreover, we sought to increase the efficiency of our time-consuming techniques for determining anion resonance energies, and demonstrate a comparatively streamlined approach that can be applied for future works.

## Experimental Section

The experimental methodology closely followed that of our previous work on U^–^(H_2_O)*_n_* anion clusters.^27,60^ A solid sample of T was heated to 220 °C within a pulsed Even-Lavie valve,^61^ backed with inert N_2_ gas (held at ∼ 5 bar) that had passed over a drop of water. The generated supersonic expansion contained thymine-water clusters, T­(H_2_O)*_n_*, to which electrons were attached using a ring filament ionizer. T^–^(H_2_O)*_n_* anions were separated by Wiley–McLaren time-of-flight mass spectrometry^62^ and intersected with laser pulses of tunable photon energy (*h*ν), generated by an Nd:YAG-pumped optical parametric oscillator. At applied photon energies above the electron binding energy of the examined anion cluster, photoelectrons were produced, the kinetic energies of which were determined by velocity map imaging.^63^ In general, outgoing electrons with less kinetic energy were directed closer to the center of the photoelectron image. Reconstruction of the photoelectron image into a photoelectron spectrum was performed using the polar onion peeling (POP) algorithm.^64^ Multiple photoelectron spectra acquired using an extensive set of photon energies were then compiled to produce a two-dimensional photoelectron spectrum.^65,66^ Photoelectron spectra were calibrated to I^–^, with a resolution ∼ 3% of the outgoing kinetic energy of the electron. In some experiments, described below, *hν* was continuously scanned, at a rate of 0.2 nm s^–1^.

## Results and Analysis

Two-dimensional photoelectron spectra, presented in Figure 1, were acquired for T^–^(H_2_O)*_n_* with *n* = 2, 3, 4, and 6. Each horizontal slice through the two-dimensional spectrum represents an individual photoelectron spectrum acquired at the specified photon energy. For each cluster, the most apparent feature in the two-dimensional spectrum is a relatively broad diagonal signal (to which the spectra are normalized), corresponding to direct photodetachment from the π_1_* ground state of T^–^(H_2_O)*_n_* to the S_0_ ground state of T­(H_2_O)*_n_*. A higher photon energy detaches electrons with an equally higher electron kinetic energy (eKE), resulting in the diagonal form of the feature. A second, less prominent diagonal feature (most noticeable in the spectra of T^–^(H_2_O)_2_) is also present, approximately 3 eV above the π_1_* → S_0_ + e^–^ signal: this arises from direct photodetachment to the lowest triplet state of the neutral cluster, π_1_* → T_1_ + e^–^.

**Figure 1:**
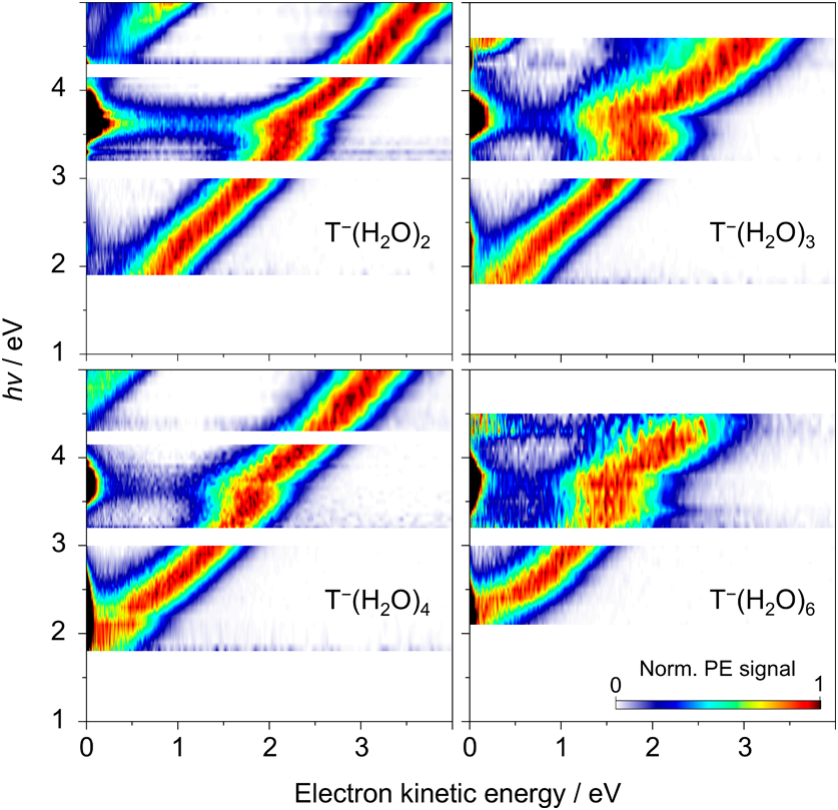
Two-dimensional photoelectron (PE) spectra for T^–^(H_2_O)*_n_*, with *n* = 2, 3, 4, and 6. Individual photoelectron spectra were acquired with photon energy spacings down to 0.05 eV and are normalized to the maximum height of the diagonal S_0_←π_1_* feature. Photoelectron spectra were not acquired at *h*ν = 3.10 and 4.20 eV (for *n* = 2 and 4) due to low laser power.

With a greater degree of microhydration (increasing *n*), the onsets of both diagonal features shift to higher photon energies. This indicates that more energy is necessary for electron detachment from a more solvated anion, and thus that hydration acts to stabilize the π_1_* state anion with respect to the neutral states, in accord with earlier studies on nucleobase-water cluster anions.^27,57^ Particularly for larger T^–^(H_2_O)*_n_*, an assortment of structural isomers (with different water binding sites to T^–^) may have been probed in our experiment, each with slightly different electron binding energies,^67^ contributing to the substantial breadth of the photoelectron signals. The overall stabilization provided by the clustered water molecules is better assessed by considering the adiabatic and vertical detachment energies (ADE and VDE, respectively) associated with each T^–^(H_2_O)*_n_* cluster. To extract these energies, additional photoelectron spectra were acquired at *h*ν = 4.70 eV for a more extensive series of T^–^(H_2_O)*_n_* anion clusters, as demonstrated in Figure 2(a). The spectra are presented in terms of electron binding energy, defined as eBE = *h*ν – eKE. The VDE is extracted from the eBE at which there is most photoelectron signal; the ADE is measured as the lowest eBE at which there is significant photoelectron signal, which we take to be 10% of the maximum. This definition for the ADE matches our previous characterizations for U^–^(H_2_O)*_n_*,^27^ aiding comparison between the different cluster species. As shown in Figure 2(b), both the ADE and VDE increase with *n*, but to a lesser extent with each successively added water molecule. While the uncertainties in the detachment energies (using typical techniques to determine the covariance matrix associated with appropriate fitting functions, for example) were generally very small, we exercise caution and report larger uncertainties of ± 0.05 eV for each VDE, and ± 0.10 eV for each ADE.

**Figure 2:**
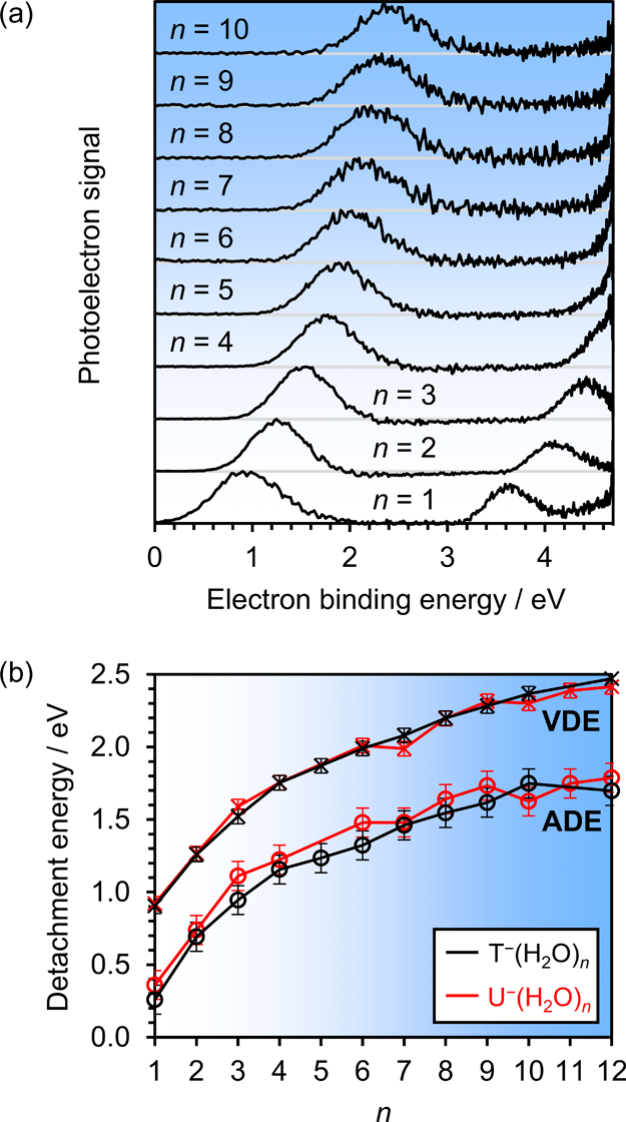
(a) Photoelectron spectra of T^–^(H_2_O)_*n* ≤ 10_, acquired with *h*ν = 4.70 eV laser pulses. (b) Adiabatic and vertical detachment energies (ADEs and VDEs, respectively) for T^–^(H_2_O)*_n_* (black) and U^–^(H_2_O)*_n_* (red) anion clusters.^27^ The estimated uncertainties in the VDEs were ± 0.05 eV, and in the ADEs were ± 0.10 eV.

The photoelectron spectra of T^–^(H_2_O)*_n_* bear striking resemblance to those previously measured for similarly sized U^–^(H_2_O)*_n_*,^27^ as may be expected from the kindred molecular structures of the T and U nucleobases. As shown in Figure 2(b), the *n*-dependent VDEs of U^–^(H_2_O)*_n_* are essentially identical to those of T^–^(H_2_O)*_n_*. There appears to be a small disparity in the ADEs, where the electron affinities of thymine-water clusters are slightly (∼0.1 eV) below their uracil analogs. Consequently, the energy gap between the ADE and the VDE is higher for T^–^(H_2_O)*_n_* than U^–^(H_2_O)*_n_*, indicating a larger anion-to-neutral reorganization energy in the case of thymine.

In the two-dimensional photoelectron spectra of T^–^(H_2_O)*_n_* presented in Figure 1, off-diagonal features were also observed. For each cluster, low-eKE electrons were generated following irradiation, particularly at photon energies 3.4 ≤ *h*ν ≤ 4.0 eV. The corresponding electron kinetic energies approximately followed a Boltzmann distribution, as shown in Figure 3(a), which is the characteristic photoelectron eKE distribution arising from (anionic) thermionic emission processes.^68–70^ Such low-energy electrons are produced when the initial anion cluster is photoexcited to a higher electronic state, and then undergoes nonadiabatic relaxation to the electronic ground state, producing a vibrationally ‘hot’ cluster susceptible to thermal (statistical) electron loss.^71,72^ Therefore, the observation of thermionic emission necessitates that an excited anion resonance is accessible (from the π_1_* state) via the absorption of 3.4 ≤ *h*ν ≤ 4.0 eV photons. Dynamics associated with the resonance are further evidenced by a deviation in the diagonal behavior of the π_1_* → S_0_ + e^–^ feature in this wavelength range, caused by autodetachment from the anion resonance that results in electrons with slightly lower kinetic energies.^66,73^

**Figure 3:**
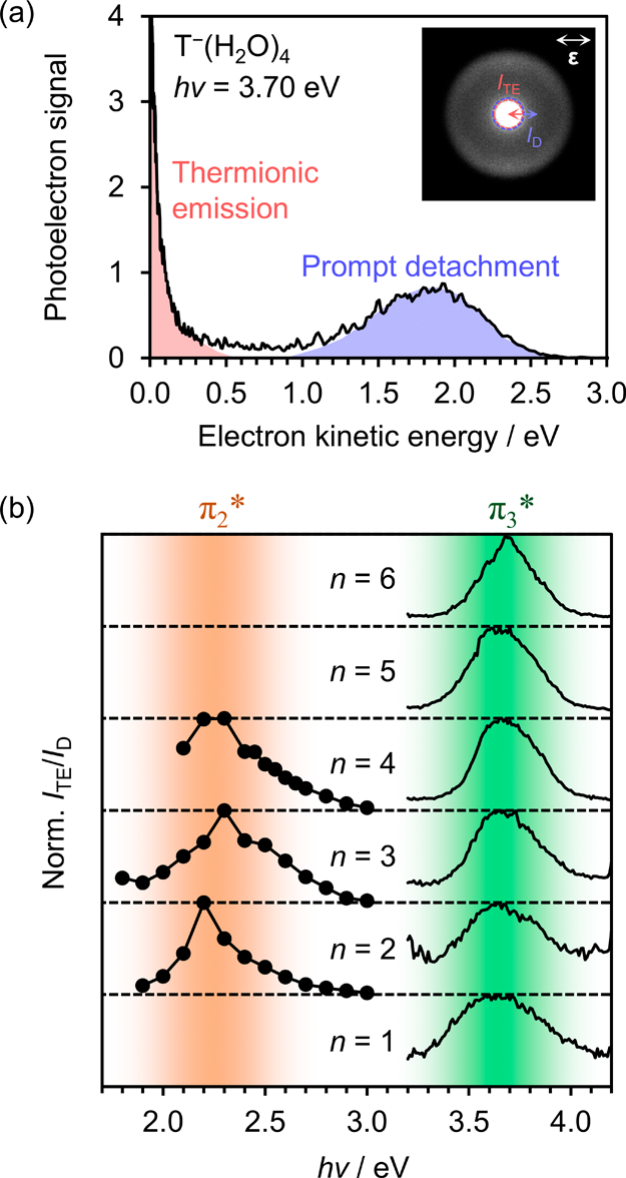
(a) Photoelectron spectrum of T^–^(H_2_O)_4_ acquired at *h*ν = 3.70 eV, resonant with the π_3_* excited state. Photoelectron signal arising from thermionic emission is highlighted in red, and prompt detachment in blue. The corresponding photoelectron image is inset, where the thermionic emission feature saturates the color scale. A red dashed circle indicates a 50 pixel radius about the center of the image. (b) Normalized ratio of photoelectron signal associated with thermionic emission (*I*_TE_) to direct detachment (*I*_D_) for T^–^(H_2_O)*_n_* anion clusters.

The electronic action spectrum associated with excitation to the resonance can be measured by tracking the amount of thermionic emission with changing photon energy.^56,74^ As the yield of thermionic emission is also dependent on the photon flux and on the ion density, quantifying the amount of thermionic emission must be performed carefully. In earlier studies, we have quantified the degree of thermionic emission by comparing the integrated photoelectron signal associated with thermionic emission to the integrated signal of a direct detachment feature in the spectrum, for each measured photon energy.^27,56,75^ For example, in the context of the spectrum shown in Figure 3(a), the amount of thermionic emission could be quantified by taking a quotient of the integral of the red shaded region (thermionic emission) by the integral of the blue shaded region (prompt detachment). This approach circumvents the photon flux and ion density dependencies, but requires a two-dimensional photoelectron spectrum to be acquired for each anion cluster, and is therefore inherently time-consuming and rather coarse in *hν* steps. A more efficient approach with potentially higher resolution is presented here: the photon energy was continuously scanned while a photoelectron action spectrum was acquired. Since low-energy electrons are velocity-mapped toward the center of the photoelectron image, the photoelectron signal associated with thermionic emission, *I*_TE_, was approximated as the total signal within a 50 pixel radius at the center of the measured photoelectron image (see Figure 3(a) inset). The photoelectrons in this region primarily corresponded to electrons with eKE < 0.1 eV. Photoelectron signal associated with directly (promptly) detached electrons, *I*_D_, was approximated as the remaining total signal in the photoelectron image, as shown inset in Figure 3(a). The resulting ratio of *I*_TE_/*I*_D_ was found to yield very similar values to our previous approach^27^ of integrating the two features in each fully acquired photoelectron spectrum (see Supporting Information for comparison), and afforded a real-time action spectrum that was essentially continuous in *h*ν. With the benefit of faster acquisition, photoelectron action spectra were also measured for two clusters for which the full frequency-resolved photoelectron spectroscopy was not performed: T^–^(H_2_O)_1_ and T^–^(H_2_O)_5_. Figure 3(b) displays the measured ratio *I*_TE_/*I*_D_ for the full series of clusters T^–^(H_2_O)_1–6_, where a single, broad peak is present within the UV region for each cluster. Moreover, the peak position is not shifting with the increasing degree of hydration, with vertical excitation energy VEE = 3.65 ± 0.10 eV, irrespective of *n*. This allows us to correlate the resonance being populated with the π_3_* anion resonance of T^–^, found in earlier electron transmission spectroscopic studies.^59^ Altogether, for the structural isomers of T^–^(H_2_O)*_n_* that were probed within our experiment, the different shape resonances of T^–^ appear to be similarly stabilized with hydration, which shows some contrast with scattering calculations on microhydrated thymine.^76^

Thermionic emission was also observed at visible wavelengths, *h*ν < 2.8 eV. At these lower photon energies, directly detached electrons are less separable from low-energy electrons arising from thermionic emission (particularly for the larger clusters), complicating the action spectroscopy. Therefore, in the visible region, we resort to our earlier approach^27^ of quantifying *I*_TE_ and *I*_D_ by the integrals of the (fitted) photoelectron spectral features corresponding to thermionic emission and direct detachment, respectively. Analysis of the T^–^(H_2_O)_6_ cluster was omitted as the direct detachment was not clearly distinguishable from thermionic emission across the visible range of *h*ν. Figure 3(b) shows a second broad and unshifting peak in the visible range, with vertical excitation energy VEE ≈ 2.25 eV. We assign the anion resonance being accessed as the π_2_* state, based again on earlier electron scattering studies.^59^ The thermionic emission associated with excitation to the π_2_* resonance appears to be more prominent for the larger T^–^(H_2_O)*_n_* clusters. This may be due to the additional vibrational degrees of freedom that are available in the larger clusters, aiding intramolecular vibrational redistribution that precedes thermionic emission.^56^

Finally, we note the asymmetric shape of the π_1_*→π_3_* transition. While this was partially observable in U^–^(H_2_O)*_n_*, as highlighted in Figure 4, it is now far clearer in the action spectrum of T^–^(H_2_O)*_n_*. The asymmetry indicates that the spectral width is arising from a combination of lifetime broadening, heterogeneous broadening, and a Franck–Condon profile. As the π_3_* resonance is a shape resonance, it may be expected to have a short autodetachment lifetime (typically on the order of tens of femtoseconds). Nevertheless, dynamics can clearly take place on the resonance, ultimately leading to reformation of the ground electronic π_1_* state. The observation that the spectral width is not increasing significantly with increasing *n* indicates that the heterogeneous broadening contribution is not that dramatic and that there is a relatively large change in geometry between the π_1_* state and the π_3_* resonance.

**Figure 4:**
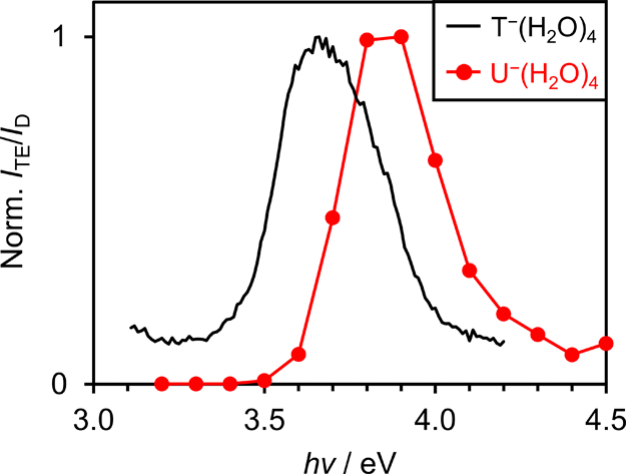
Normalized ratio of photoelectron signal associated with thermionic emission (*I*_TE_) to direct detachment (*I*_D_) for the T^–^(H_2_O)_4_ and U^–^(H_2_O)_4_ anion clusters.^27^

## Discussion

U^–^(H_2_O)*_n_* clusters also exhibited photoexcitation to their π_3_* resonances with UV light,^27^ although the corresponding VEE differed to that of T^–^(H_2_O)*_n_*. Taking the *n* = 4 clusters for example, the relative thermionic emission yields are shown in Figure 4, demonstrating that the π_1_*→π_3_* transition is ∼ 0.2 eV lower in energy for T^–^(H_2_O)*_n_* than for U^–^(H_2_O)*_n_*. This ordering is somewhat surprising, as electron transmission spectroscopy of the isolated nucleobases revealed the π_1_* and π_3_* resonances to be closer in energy for U than for T.^59^ However, our anion photoelectron spectroscopy experiment probes the relative resonance energies within the equilibrium geometry of the ground-state anion, rather than in the neutral geometry pertinent to electron attachment. This anion-to-neutral reorganization energy may slightly differ between the π_1_* and π_3_* states, for T and for U, resulting in this different ordering. The lower VEE associated with T^–^(H_2_O)*_n_* may also indicate some mixed excitation character with a nearby Feshbach resonance, which has been predicted to be lower in energy for T^–^ than for U^–^.^77^ Photoexcitation to the π_2_* resonance was less clearly quantifiable in the earlier case of the U^–^(H_2_O)*_n_* clusters as it is in the present study, making a direct comparison with thymine more difficult.

Our earlier experiments on U^–^(H_2_O)*_n_* reached up to *n* = 35, allowing the corresponding VDEs, ADEs, and resonance energies to be extrapolated to the bulk aqueous limit (*n* = ∞). Given that the VDE and ADE of T^–^(H_2_O)*_n_* follow those of U^–^(H_2_O)*_n_* very closely (at least up to *n* = 12), and that the π_1_*→π_3_* excitation energies are unchanging with cluster size, it appears that similar extrapolations and analyses can be performed on the thymine-water cluster anions presented in the current study. Here, we assume that the VDEs of T^–^(H_2_O)*_n_* follow the extrapolated values for U^–^(H_2_O)*_n_*, which is justifiable based on the close correlation in their respective VDEs shown in Figure 2(b). This analysis yields VDE­(T^–^_(aq)_) = 4.8 ± 0.3 eV and ADE­(T^–^_(aq)_) = 3.4 ± 0.3 eV, and thus the reorganization energy λ_(aq)_ ≈ 1.4 eV (compared to ∼ 1.2 eV for U_(aq)_).

Using the extrapolated energies above and within linear response theory,^78^ an approximate Marcus diagram for T^–^_(aq)_/T_(aq)_ can be constructed and compared to our earlier schematic for uracil,^27^ as shown in Figure 5. Due to the greater observed reorganization energy λ_(aq)_ associated with electron attachment to thymine than to uracil, the potential energy surfaces are expected to be slightly steeper for thymine. In the equilibrium geometry of anionic T^–^_(aq)_, denoted *Q*_anion_, the π_3_* resonance is expected to be vertically bound with respect to electron loss (i.e., a bound electronic state). However, for T_(aq)_ within its natural equilibrium geometry, *Q*_neutral_, the π_3_* state is expected to be a resonance and therefore accessible via electron attachment with an approximate vertical electron attachment energy VAE_(aq)_ ≈ 1.6 eV. Following electron attachment into the π_3_* resonance of aqueous thymine, spontaneous solvent reorganization will rapidly act to stabilize the generated anion into a vertically bound state.^79,80^ Furthermore, as evidenced by the observation of thermionic emission from (especially the larger) T^–^(H_2_O)*_n_* clusters, internal conversion can follow to form the ground state of the anion (π_1_*). Similar attachment dynamics are expected to occur into the π_2_* resonance of T_(aq)_, which appears to be accessible with very low-energy incident electrons. The π_2_* resonance becomes an adiabatically stable state following little solvent relaxation, further enhancing the propensity for formation of a stable anion.

**Figure 5:**
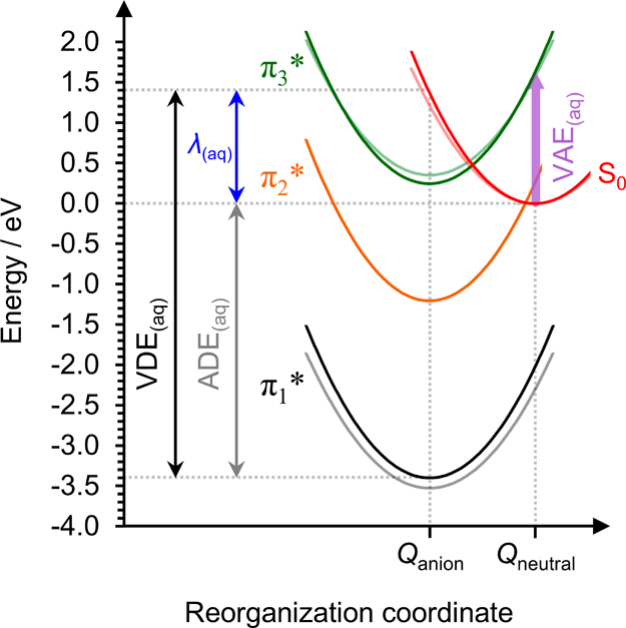
Schematic Marcus diagram of aqueous thymine (opaque lines), overlaid onto the earlier diagram determined for uracil^27^ (faded lines). Aqueous vertical and adiabatic detachment energies (VDE_(aq)_ and ADE_(aq)_, respectively) are displayed, along with their difference – the reorganization energy, λ_(aq)_.

The above arguments that allow us to estimate the resonance positions of the *neutral* in aqueous solution, however, should be cautioned as they are based on the assumptions that the reorganization coordinate is harmonic and that *Q*_anion_ is the same for π* states, which we have shown not to be accurate based on the spectral line shape highlighted in Figure 4. However, the dominant contribution to the reorganization energy of the π_1_* state is due to hydration (outer-sphere),^81^ which is likely to also be true for the π_2_* and π_3_* resonances. It is also important to note that electron scattering resonances for U_(aq)_ will have various other factors to consider such as the lifetime of the TNI, induced polarization of the water and its effect (both temporally and energetically) on the TNI, and polaron formation dynamics; none of these are considered in the current pictures. Finally, we comment that the electron capture dynamics through the π_2_* and π_3_* resonances differ to those of the hydrated electron, where we have recently shown how the water molecules define the reorganization coordinate.^82^

## Conclusions

Frequency-resolved photoelectron spectra of T^–^(H_2_O)*_n_* anion clusters were acquired to elucidate the energetic positions of the π_1_*, π_2_* and π_3_* resonances of T^–^ with an increasing degree of hydration. Each π* resonance was stabilized similarly, and the exhibited behavior closely reflected the hydration-induced stabilization of the corresponding resonances of U^–^.^27^ For each cluster, the vertical detachment energy of the anion ground state (π_1_*) of T^–^(H_2_O)*_n_* was measured to be essentially identical to U^–^(H_2_O)*_n_*, enabling an extrapolation of the resonance energies to the bulk aqueous limit to be performed. However, the vertical excitation energy between the π_1_* and π_3_* states was approximately 0.2 eV smaller for T^–^_(aq)_ than U^–^_(aq)_. Therefore, assuming that the electron attachment energies into the π_3_* resonances of T_(aq)_ and U_(aq)_ are similar, the π_3_* state of T^–^_(aq)_ becomes vertically bound to a greater extent upon geometric relaxation of the surrounding solvent sphere, which would enhance the long-term electron capture efficiency for this hydrated nucleobase compared to uracil. Overall, our results demonstrate that low-energy electron attachment into both the π_2_* and π_3_* resonances of the T nucleobase are likely to be accessible in aqueous solution (i.e., they are resonances in the geometry of the neutral), with the latter lying at a vertical electron attachment energy, VAE_(aq)_ ≈ 1.6 eV. This represents a fundamental step in identifying exactly which anion resonance states participate most in electron attachment to the nucleobase moieties of DNA.

## Supplementary Material



## Notes

Quantification of thermionic emission (PDF)
