# Supplementary material for: The Influence of Water Molecules on the π* Shape Resonances of the Thymine Anion
Source: J Phys Chem A. 2025 May 12;129(26):5771–8. doi: 10.1021/acs.jpca.5c01948 (PMC12235612; doi:10.1021/acs.jpca.5c01948)
Supplement: Supplementary file 1 [file jp5c01948_si_001.pdf]

Supporting information for:

The Influence of Water Molecules on the π* Shape Resonances of the Thymine Anion

Connor J. Clarke[1], E. Michi Burrow[1], Jan R. R. Verlet[1,2]*

[1]*Department of Chemistry, Durham University, Durham DH1 3LE, United Kingdom*

[2]*J. Heyrovský Institute of Physical Chemistry, Czech Academy of Sciences, Dolejškova 3, 18223 Prague 8, Czech Republic*

*Email: j.r.r.verlet@durham.ac.uk

Quantification of thermionic emission

As mentioned in the main manuscript, the amount of thermionic emission at different excitation wavelengths was quantified by comparing the photoelectron signal corresponding to thermionically emitted electrons, I_{TE}, to the photoelectron signal corresponding to directly detached electrons, I_D. We applied two different techniques to measure I_{TE} and I_D, and a comparison is presented below.

In our original method for finding I_{TE}/I_D,[1] we measured each I as the integral of the corresponding feature in the photoelectron spectrum, at the discrete set of photon energies used to construct the two-dimensional photoelectron spectrum. An example at one photon energy for one cluster ($T^{-}(H_2O)_4$ using hv = 3.7 eV) is shown in Figure S1(a), where the integrals are represented by the shaded regions. This provides one data point in Figure S1(c), for the data set labelled 'original' (black). Calculating I_{TE}/I_D at different photon energies then reveals the location of the anion resonance.

In our new method, I_{TE} was approximated as the summed intensity of each pixel of the photoelectron image within a 50 pixel radius of the center, as displayed in Figure S1(b). I_D was approximated as the remaining summed intensity of the image. With sufficient total photoelectron signal, I_{TE}/I_D could be measured on a shot-to-shot basis (and a laser repetition rate of 10 Hz) in the new approach. Thus, we could utilize the wavelength-scanning feature of our optical parametric oscillator to acquire a full action spectrum (see Figure S1(c), labelled 'new' and in blue) within a few minutes. This was far faster than the original approach, which typically required many hours of measurement and yielded a sparser set of I_{TE}/I_D. Overall, the set of I_{TE}/I_D exhibited excellent agreement between the two approaches.

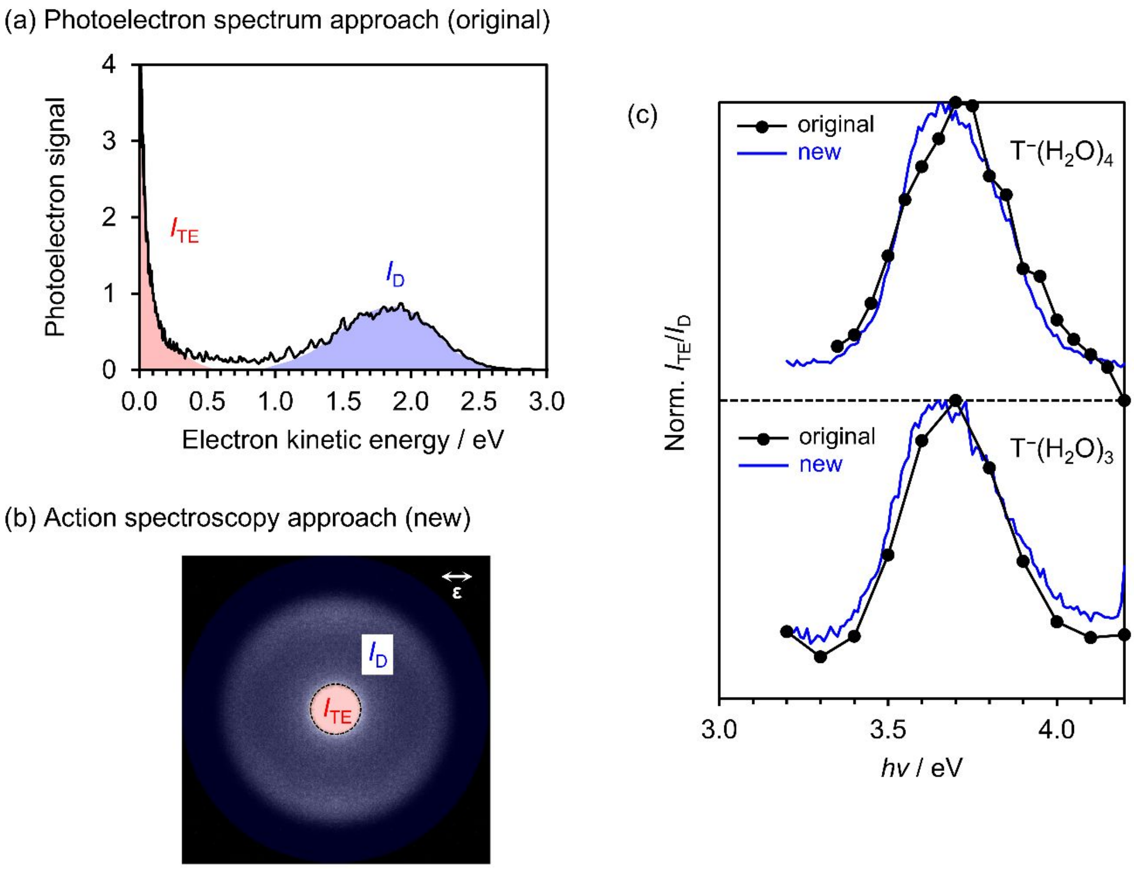

Figure S1: Example determination of I_{TE}/I_D in (a) the original photoelectron spectrum approach, where the colored regions represent the calculated integrals; and (b) the new action spectroscopy approach, where the colored regions represent the areas over which the intensity was summed. (c) Comparison between the two approaches described above for two clusters, $T^-(H_2O)_3$ and $T^-(H_2O)_4$, acquired with UV laser pulses.

References

1 G. A. Cooper, C. J. Clarke and J. R. R. Verlet, *J. Am. Chem. Soc.*, 2023, **145**, 1319–1326.
